# New Episodes and Suicidal Risks in Bipolar and Major Depressive Disorder Patients During Versus Before Long‐Term Treatment With Lithium

**DOI:** 10.1111/acps.70002

**Published:** 2025-07-01

**Authors:** Maurizio Pompili, Isabella Berardelli, Salvatore Sarubbi, Elena Rogante, Mariarosaria Cifrodelli, Denise Erbuto, Dorian A. Lamis, Ross J. Baldessarini

**Affiliations:** ^1^ Department of Neurosciences Mental Health and Sensory Organs, Suicide Prevention Centre, Sant'andrea Hospital, Sapienza University of Rome Rome Italy; ^2^ International Consortium for Bipolar & Psychotic Disorders Research, Mailman Research Center, McLean Hospital Belmont Massachusetts USA; ^3^ Department of Human Neurosciences Sapienza University of Rome Rome Italy; ^4^ Department of Psychiatry and Behavioral Sciences Emory University School of Medicine/Grady Health Systems Atlanta Georgia USA; ^5^ Department of Psychiatry Harvard Medical School Boston Massachusetts USA

**Keywords:** before vs. during treatment, bipolar disorder, lithium, long‐term treatment, major depressive disorder

## Abstract

**Objectives:**

Lithium treatment reduces the risk of recurring episodes in bipolar disorder (BD) and probably also in major depressive disorder (MDD) and has evidence of antisuicidal effects. Study objectives were to test for effects of adding lithium treatment for one year to a year of other treatments on risks of illness recurrence, suicidal ideation, and suicide attempts.

**Methods:**

We compared 296 major mood disorder outpatients for 12 months with treatment that did not include lithium versus 12 months with lithium included. We considered differences in the recurrence of new episodes of illness, new suicidal ideation and suicide attempts, and estimated time to these outcomes with survival analyses.

**Results:**

With lithium treatment included, there were marked reductions in episode recurrences (3.12‐fold), suicidal ideation (4.78‐fold), and suicide attempts (6.54‐fold) in both BD and MDD patients, with corresponding delays to these outcomes.

**Conclusions:**

Adding lithium treatment was strongly associated with reduced risk and delay of clinical recurrence, suicidal ideation and suicide attempts in both BD and MDD outpatients.

**Summary**

- Significant outcomes
  ○ Adding lithium to treatment for one year reduced risk of illness recurrence by > 3‐fold and increased latency to new episodes in both bipolar and major depressive disorder patients.
  ○ In addition, the risk of new suicidal ideation and attempt was reduced by 4.78‐ and 6.59‐fold, respectively, and the latency to these outcomes was significantly increased.
  ○ The findings add support for the broad utility of lithium treatment in major affective disorders.
- Limitations
  ○ Sample size was limited, especially for major depressive disorder.
  ○ Observations were naturalistic and unblinded.
  ○ Contributions of treatments other than lithium were not determined.
  ○ Findings may not generalize to other settings.

## 1 Introduction

Lithium is widely used as a mood‐stabilizing agent to prevent illness recurrences in patients with bipolar disorder (BD) as well as for unipolar major depressive disorder (MDD) [1, 2, 3, 4, 5, 6]. Long‐term naturalistic studies and randomized controlled trials consistently support the efficacy of lithium in reducing episode recurrence and preventing hospitalization in both BD and MDD populations, typically for 6–12 months [7, 8, 9]. Therapeutic benefits of lithium also include augmentation of responses in treatment‐resistant depression [10, 11] as well as management of acute manic episodes [12]. Meta‐analyses indicate that lithium maintenance treatment has a greater effect in preventing manic than depressive recurrences in BD [7, 13]. In addition, lithium has evidence of antisuicidal effects in major affective disorder patients that is not associated with other mood‐stabilizing agents, as well as the ability to counter aggressive behavior generally [4, 5, 6, 7, 8, 9, 10, 11, 12, 13, 14, 15, 16, 17, 18, 19, 20, 21]. Discontinuation of lithium treatment in BD patients, particularly rapidly, can precipitate episodes of affective illness [22, 23, 24] and increase suicidal risk [17, 20, 25] as well as yielding other clinical, social, and economic burdens [26, 27]. These findings underscore the clinical value of maintaining long‐term treatment with therapeutic circulating levels of lithium [28].

Despite strong evidence of lithium's therapeutic effects in major mood disorders, questions remain regarding specific rates and timing of new episodes and suicidal behavior during lithium treatment [20, 29], particularly in naturalistic settings [30]. Better understanding of such details should contribute to optimizing treatment of mood disorder patients [12, 31] by identifying high‐risk periods [31] and their timing [21, 32]. Such a study reported recently by Pompili et al. [21] found support for lithium's effectiveness in preventing rehospitalization of BD and MDD patients by comparing hospitalization rates during 12 months before starting lithium (40.4%) versus during 12 months of its included (11.2%; 3.61‐fold reduction), with similar benefits with both diagnoses.

## 2 Aims of the Study

We aimed to estimate survival probabilities for clinical recurrence, suicidal ideation, and suicide attempt in outpatients diagnosed with Bipolar Disorder or Major Depressive Disorder in 12‐month periods before versus during treatment that included lithium carbonate. We hypothesized that adding lithium treatment would reduce risks of (a) clinical recurrence, (b) suicidal ideation and (c) suicide attempts, and (d) we tested for diagnosis‐selective effects.

## 3 Methods

### 3.1 Participants

The study sample included 296 consenting adult psychiatric outpatients (162 women, 134 men; mean age ± SD = 44.1 ± 12.8 years; range = 18–74 years) enrolled at the Psychiatric Unit of Sant'Andrea Hospital in Rome, Italy. Part of this sample was included in a previous study on risk of hospitalization [21]. Inclusion criteria were: (1) age ≥ 18 years, (2) DSM‐5‐TR diagnosis of either bipolar disorder (BD) or major depressive disorder (MDD), and (3) ability to provide written informed consent. Patients were excluded if they had comorbid neurological conditions (including dementia, Parkinson's disease, or epilepsy), cognitive impairment, language barriers that could interfere with assessment, or were unwilling or unable to provide informed consent. The study protocol followed ethical principles of the Declaration of Helsinki and received approval from the hospital institutional ethical review board (review CE‐6626/2021). All participants received detailed information about study procedures and provided written informed consent prior to enrollment.

### 3.2 Measures

Demographic and clinical data were collected using a structured history form, with information including sex, age, and diagnosis. All psychiatric diagnoses were established according to the APA *Diagnostic and Statistical Manual of Mental Disorders*, fifth edition, text revision (DSM‐5‐TR) [33] by experienced academic psychiatrists. Suicidal ideation and attempts were assessed by experienced psychiatrists specifically trained in using standardized definitions including criteria specified in the Columbia‐Suicide Severity Rating Scale (C‐SSRS) [34, 35, 36]. Suicide attempts were defined as proposed by Silverman et al. [37, 38] as nonfatal, self‐directed, potentially injurious behaviors accompanied by intent to die, regardless of resulting injury. Clinical “recurrence” was defined as the appearance of a new syndromal episode of [hypo]mania or major depression meeting DSM‐5‐TR criteria [39, 40]. This definition was applied consistently across both BD and MDD diagnoses to evaluate worsening symptoms, timed at their first appearance. During treatment with lithium, other psychotropic medicines were given, as reported below (Table 1). Enrolled patients and clinical evaluators were not blinded to treatments given.

**TABLE 1:** Characteristics of 296 study subjects.

All subjects (*n* = 296)	% or Mean [±95% CI]
*Sex (%)*	
Women (*n* = 162)	54.7 [52.2–57.1]
Men (*n* = 134)	45.3 [42.6–48.0]
Current age (years)	44.1 [42.6–45.6]
*Diagnosis*	
Bipolar disorder (*n* = 171)	57.8 [53.4–60.1]
Major depressive disorder (*n* = 125)	42.2 [41.3–43.1]
Age at illness onset (years)	30.8 [29.5–32.1]
Untreated illness (months)	21.2 [16.6–25.8]
*Baseline rates (%)*	
Current suicidal ideation	42.2 [40.4–44.0]
Suicide attempt in ≤ 6 months	17.2 [15.9–18.6]
*Other treatments (%)*	
Antipsychotics	78.0 [76.5–79.5]
Benzodiazepines	57.4 [55.6–59.2]
Mood‐stabilizing anticonvulsants	47.3 [41.5–53.2]
Antidepressants	36.5 [31.0–42.3]
Lithium carbonate (mg/day)	605 [575–635]

Bipolar disorder patients (*n* = 177)	% or Mean [±95% CI]
Sex	
Male	45.0 [37.4–52.8]
Female	55.0 [47.2–62.6]
Age at illness onset	29.7 [28.3–31.1]
Untreated illness (months)	17.2 [11.3–23.1]
*Baseline rates (%)*	
Current suicidal ideation	31.0 [24.2–38.5]
Recent suicide attempt	13.5 [11.9–15.2]
*Other treatments (%)*	
Antipsychotics	84.8 [78.5–89.8]
Mood‐stabilizing anticonvulsants	52.6 [51.9–53.4]
Benzodiazepines	52.0 [49.6–54.4]
Antidepressants	22.8 [22.2–23.4]
Lithium carbonate (mg/day)	626 [587–665]

Major depressive disorder patients (*n* = 125)	% or Mean [±95% CI]
*Sex (%)*	
Women	54.4 [45.3–63.3]
Men	45.6 [36.7–54.7]
Age at illness onset (years)	34.1[31.8–36.4]
Untreated illness (months)	26.6 [19.2–34.0]
*Baseline rates (%)*	
Current suicidal ideation	57.6 [48.4–66.4]
Suicide attempt in ≤ 6 months	22.4 [15.4–30.7]
*Other treatments (%)*	
Antipsychotics	68.8 [59.9–76.8]
Benzodiazepines	64.8 [55.8–73.1]
Antidepressants	55.2 [46.5–64.1]
Mood‐stabilizing anticonvulsants	40.0 [31.3–49.1]
Lithium carbonate (mg/day)	576 [529–623]

### 3.3 Data Analysis

There were 296 observations for the pretreatment and during‐lithium‐treatment phases, with one observation/patient for each phase. Cox proportional hazards regression modeling was used to evaluate the association between predictor variables and time‐to‐event (onset of a new episode of illness or new suicidality). The modeling estimated hazard ratios (HRs) for each covariate, quantifying their effect on the likelihood of experiencing the event over time, while adjusting for other factors. Prior to model fitting, we tested the proportional hazards assumption by including time‐dependent covariates for each predictor variable. A nonsignificant *p*‐value (> 0.05) for the time interactions confirmed that the proportional hazards assumption held, allowing for consistent hazard ratios over time.

A paired‐sample proportions analysis was first conducted to compare the pre‐ and during‐lithium treatment event rates for clinical recurrence, suicidal ideation and suicide attempt. The mid‐*p*‐adjusted binomial test was used to assess the statistical significance of differences in proportions.

We also performed Kaplan–Meier survival analyses to estimate survival probabilities and latencies for the three outcome types. Separate analyses were conducted to compare survival curves for each event type between the 12‐month pre‐ and during‐lithium treatment phases. The log‐rank test was used to evaluate statistical differences between the survival curves to assess the impact of lithium treatment on time‐to‐event distributions.

We also tested for the effects of adding lithium treatment in the diagnostic groups considered separately. These analyses included the use of the Log‐Rank test to assess differences in Kaplan–Meier survival curves between BD and MDD patients, including times to new depressive versus [hypo]manic episodes. In addition, chi‐square tests of independence were used to evaluate whether the diagnostic category (BD vs. MDD) was significantly associated with improvement in recurrence, suicidal ideation, and suicide attempts. Finally, to examine whether changes in suicidal symptoms were related to changes in mood recurrence, further chi‐square analyses compared improvement in recurrence risk and improvement in suicidal ideation or suicide attempts. Analyses were conducted using IBM‐SPSS.30 commercial statistical software.

## 4 Results

### 4.1 Sample Characteristics

Characteristics of the overall sample (*n* = 296) and of those diagnosed with BD (*n* = 171) or MDD (*n* = 125) are summarized in Table 1. Mean age (±SD) at illness onset was 30.8 ± 11.2 years. At study entry, 125 patients (42.2%) reported experiencing current suicidal ideation, and 51 (17.2%) had attempted suicide within six months of intake. At the start of lithium treatment, the daily nadir of serum lithium concentration averaged 0.71 (SD = ±0.26) mEq/L at a mean daily dose of lithium carbonate of 605 ± 262 mg. Overall, other psychotropic medicines given included antipsychotics (78.0%), benzodiazepines (57.4%), mood‐stabilizing anticonvulsants (47.3%), and antidepressants (36.5%), as reported in Table 1 for BD and MDD all subjects and separately for those with BD or MDD.

Age at illness‐onset averaged 29.7 ± 9.43 years among BD patients (Table 1). At study entry, 53/171 BD patients (31%) reported ever experiencing suicidal ideation, and 23 (13.5%) had attempted suicide within six months. During the 12‐months of lithium treatment, other treatments selected clinically for BD patients included antipsychotics (84.8%), mood‐stabilizing anticonvulsants (52.6%), benzodiazepines (52.0%), and antidepressants (22.8%). Among MDD patients age of onset averaged 32.4 ± 13.1 years. At study entry, 72/125 MDD patients (57.6%) reported suicidal ideation, and 28 (22.4%) had made a suicide attempt within the previous six months. During the 12‐months of lithium treatment, other treatments selected clinically for MDD patients included antipsychotics (68.8%), benzodiazepines (64.8%), antidepressants (55.2%), and mood‐stabilizing anticonvulsants (40.0%; Table 1).

### 4.2 Cox Regression

We used Cox regression to assess whether gender or diagnosis influenced the likelihood of experiencing a new episode of illness. We first checked the proportional hazards assumption by adding an interaction between time and the predictor variables (gender and diagnosis) in the model. This test showed no significant improvement with the interaction included (*p*‐value = 0.432), indicating that the hazard ratios can be considered constant over time. Neither gender nor diagnosis had a statistically significant effect on the likelihood of experiencing an illness recurrence (both *p* ≥ 0.40).

### 4.3 Effects of Lithium Treatment

We examined the association of lithium treatment with three outcomes: clinical recurrence, suicide attempt, and new suicidal ideation, using a paired‐sample approach. The primary objective was to evaluate whether there was a statistically and clinically significant reduction in the proportion of patients experiencing the specified adverse events during treatment with lithium Table 2 presents an analysis of paired‐sample proportions for the three clinical outcomes of interest based on comparing the years before versus during lithium treatment, showing large reductions in all three adverse outcomes during treatment with lithium, with similar effects in both diagnostic subgroups.

**TABLE 2:** Risk of adverse outcomes before versus during lithium treatment included.

Diagnosis	Before Lithium	With Lithium	Rate Ratio	*p*‐value [χ^2^]
***Recurrence***				
All cases [95% CI]	97/296 = 32.8% [27.6–38.3]	31/295 = 10.5% [7.40–14.4]	3.12	< 0.0001 (43.2)
Bipolar disorder [95% CI]	53/171 = 31.0% [24.4–38.2]	17/171 = 9.90% [6.10–15.1]	3.13	< 0.0001 (23.3)
Major Depressive Disorder [95% CI]	44/125 = 35.2% [27.2–43.8]	14/125 = 11.2% [6.60–7.60]	3.14	< 0.0001 (20.2)
***Suicidal Ideation***				
All cases [95% CI]	143/296 = 48.3% [42.7–54.0]	30/296 = 10.1% [7.10–14.0]	4.78	< 0.0001 (104)
Bipolar disorder [95% CI]	62/171 = 36.3% [29.3–43.6]	13/171 = 7.60% [4.11–12.6]	4.78	< 0.0001 (40.0)
Major Depressive Disorder [95% CI]	81/125 = 64.8% [56.2–72.8]	17/125 = 13.6% [8.40–20.4]	4.76	< 0.0001 (68.7)
***Suicide Attempt***				
All cases [95% CI]	85/296 = 28.7% [23.8–34.1]	13/296 = 4.39% [2.50–7.20]	6.54	< 0.0001 (49.8)
Bipolar disorder [95% CI]	37/171 = 21.6% [16.0–28.3]	5/171 = 2.90% [1.10–6.30]	7.45	< 0.0001 (27.8)
Major Depressive Disorder [95% CI]	48/125 = 38.4% [30.2–47.1]	8/125 = 6.40% [3.10–10.7]	6.00	< 0.0001 (36.8)

#### 4.3.1 Clinical Recurrence

During the pre‐lithium‐treatment period, there were 296 observations, with 97 adverse clinical events (32.8%) and a censoring rate of 67.2%. In the during lithium‐treatment period, there were 295 observations, with 31 events (10.5%) and a censoring rate of 89.5%. These results represent a reduction in the rate of events in the during‐treatment period compared to the pretreatment period by 3.12‐fold (32.8/10.5)—a major benefit of lithium treatment (Table 2). The estimated mean survival time until clinical recurrence in the pretreatment phase was 10.3 [95% CI: 9.92–10.6] months; with lithium treatment, mean survival was 11.4 [11.2–11.6] months. These survival times differed modestly (1.11‐fold) but highly significantly (by log‐rank test, *p* < 0.001), favoring lithium treatment (Table 3).

**TABLE 3:** Months to adverse outcomes before versus during lithium treatment.

Diagnosis	Months Before Lithium	Months With Lithium	Latency Ratio	*p‐*value [χ^2^]
***New Illness Episode***				
All cases [95% CI]	10.3 [9.92–10.6]	11.4 [11.2–11.6]	1.11	< 0.001 42.71
Bipolar disorder [95% CI]	10.4 [9.96–10.8]	11.5 [11.2–11.7]	1.10	< 0.001 23.10
Major Depressive Disorder [95% CI]	10.1 [9.55–10.6]	11.4 [11.0–11.7]	1.14	< 0.001 19.55
***Suicidal Ideation***				
All cases [95% CI]	9.29 [8.91–9.68]	11.4 [11.2–11.6]	1.23	< 0.001 102.80
Bipolar disorder [95% CI]	10.1 [9.65–10.5]	11.6 [11.3–11.8]	1.15	< 0.001 40.48
Major Depressive Disorder [95% CI]	8.22 [7.58–8.85]	11.2 [10.8–11.6]	1.36	< 0.001 67.73
***Suicide Attempt***				
All cases [95% CI]	10.3 [9.94–10.6]	11.8 [11.6–11.9]	1.15	< 0.001 65.51
Bipolar disorder [95% CI]	10.8 [10.4–11.2]	11.8 [11.6–12.0]	1.09	< 0.001 27.44
Major Depressive Disorder [95% CI]	9.56 [8.93–10.2]	11.7 [11.4–12.0]	1.22	< 0.001 37.63

We also examined whether the polarity of new illness (depressive or [hypo]manic) was associated with time to clinical recurrence. There were 198 observations regarding depressive episodes, with 20 events (10.1%) and a censoring rate of 89.9%; for [hypo]manic episodes, there were 61 observations, with 10 events (16.4%) and a censoring rate of 83.6%. Across the entire dataset of 259 observations, 30 events were recorded (11.6%) with a censoring rate of 88.4%. The mean estimated survival time until clinical relapse into a depressive episode was 11.5 (CI: 11.2–11.7) months and time to a new [hypo]manic episode was 11.0 (10.4–11.6). Log‐rank test (*p*‐value = 0.175) indicated a lack of statistical difference in survival distributions between patients with new depressive versus [hypo]manic episodes during treatment with lithium.

We also calculated changes in clinical recurrence rates for times before versus during treatment that included lithium for BD and MDD patients separately (Table 2). For MDD patients, the difference in recurrence risk with versus during lithium treatment was 35.2% versus 11.2% (3.14‐fold decrease); for BD patients, this difference was 31.0% versus 9.90% (3.23‐fold decrease), indicating similar benefits of lithium treatment with both diagnostic groups (*z* = 0.430, *p* = 0.667).

#### 4.3.2 Suicidal Ideation

Overall, among 296 observations, there were 143 new suicidal events without lithium (48.3% [42.7–54.0]) versus 30 (10.1% [7.10–14.0]) with lithium added, at a censoring rate of 88.8%. These *rates* (48.3/10.1) indicate a highly significant, 4.78‐fold overall decrease with lithium added (Table 2). *Latency* to new suicidal ideation with versus before lithium treatment increased from 9.29 [8.91–9.68] to 11.4 months [11.2–11.6] (a 1.23‐fold increase; Table 3).

With MDD, the *risk* of new suicidal ideation decreased with lithium treatment from 64.8% [56.2–72.8] to 13.6% [8.40–20.4], a decrease of 4.76‐fold. With BD, these rates decreased from 36.3% [29.3–43.6] to 7.60% [4.11–12.6] (4.78‐fold reduction; Table 2). These improvements were greater with MDD than BD (*z* = 3.35, *p* < 0.001). The mean estimated *survival time* to new suicidal ideation with MDD increased from 8.22 [7.58–8.85] to 11.2 [10.8–11.6] months (an increase of 1.36‐fold). With BD cases, this latency increased similarly from 10.1 [9.65–10.5] to 11.6 [11.3–11.8] months (i.e., by 1.15‐fold; Table 3).

#### 4.3.3 Suicide Attempt

During the pretreatment period, there were 296 observations, with 85 suicidal events (28.7%) and a censoring rate of 71.3%; with lithium treatment there were 296 observations, with 13 events (4.39%) and a censoring rate of 95.6% (Table 2). These results represent a 6.54‐fold (28.7/4.39) reduction in the proportion of patients who attempted suicide during treatment with lithium—a major beneficial effect (Table 2). The mean [CI] estimated survival time to a suicide attempt was 10.3 [9.94–10.6] months before, and 11.8 [11.6–11.9] months during treatment with lithium (Table 3). This modest difference (11.8/10.3 = 1.15‐fold) was highly significant by log‐rank test (*p* < 0.001).

**FIGURE 1:**
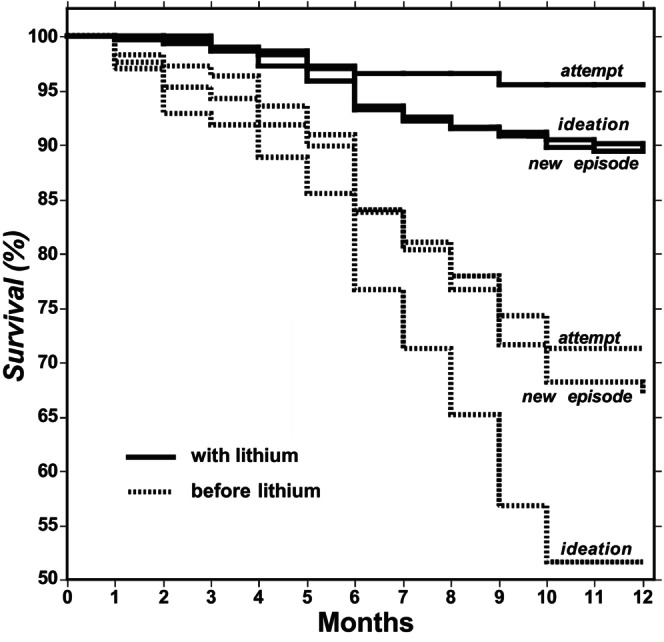
Survival functions for time to a new episode of illness, suicidal ideation or suicide attempt among 296 major affective disorder patients in one year before versus one year during treatment with lithium carbonate. All survival outcomes are highly significantly improved with lithium.

We also examined whether the primary clinical diagnosis was associated with risk of a suicide attempt (Table 2) or with time to a first attempt (Table 3). With MDD, these respective rates were 38.4% [30.2–47.1] versus 6.40% [3.10–10.7] (6.00‐fold reduction). With BD the *risk* of a suicide attempt before lithium was 21.6% [16.0–28.3] compared to 2.90% [1.10–6.30] during treatment with lithium included (7.45‐fold reduction), indicating a greater effect with BD than MDD (*z* = 2.34, *p* = 0.025). *Latency* to a suicide attempt with MDD increased from 9.56 [8.93–10.2] to 11.7 (11.4–11.9) months (1.22‐fold reduction) and with BD from 10.8 [10.4–11.2] to 11.8 [11.6–11.9] months during treatment that included lithium, a modest (1.09‐fold) difference. Survival analyses for all three outcome measures without versus with lithium treatment are summarized in Figure 1.

#### 4.3.4 Suicidal Risk and Affective Symptoms

Finally, we explored associations between persistent clinical symptoms and suicidal outcomes during a year of lithium treatment. Among patients who did not present mood symptoms at follow‐up, suicidal ideation was reported by only 2/208 patients (0.96%), and no suicide attempts were recorded. Conversely, with ongoing affective symptoms (*n* = 51), more than half (52.9%) reported suicidal ideation, and 25.5% attempted suicide. There was a highly significant association between persistent mood symptoms and suicidal ideation (Cramer's *v* = 0.64; *p* < 0.0001) as well as the risk of suicide attempt (*v* = 0.44, *p* < 0.0001). All suicide attempts occurred in patients who remained symptomatic, with no such events among asymptomatic patients.

## 5 Discussion

In this study of major mood disorder patients, we confirmed the hypothesis that adding lithium treatment would reduce risks and increase latency to clinical recurrence, new suicidal ideation, and suicide attempt by considering 12‐month periods in the same subjects before and during treatment that included lithium. Cox regression analysis indicated that neither gender nor diagnosis had a statistically significant effect on the likelihood of experiencing an illness recurrence. We used Kaplan–Meier survival analyses to estimate risks and latencies to first instances of new clinical recurrence, suicide attempt, and suicidal ideation.

Of 296 patients enrolled, 32.8% experienced an episode of clinical recurrence within the year prior to treatment with lithium versus only 10.5% during the year with lithium treatment included, indicating a 3.12‐fold reduction in the risk of recurrences of BD and MDD with lithium. In the year before adding lithium, 48.3% of patients reported new suicidal ideation, compared to only 10.1% during the year with lithium treatment, indicating a 4.78‐fold reduction. Similarly, for suicide attempt, the rate before lithium was 28.7%, compared to 4.39% during the year with lithium included, indicating a 6.54‐fold reduction with lithium.

The marked reductions in illness recurrence, suicidal ideation, and suicide attempts with longer latencies to these outcomes observed among both BD and MDD (Tables 2 and 3) patients extend previous evidence supporting lithium's efficacy in mood stabilization and suicide prevention in major mood disorder patients [4, 20, 21, 41, 42, 43, 44, 45, 46]. Moreover, this appears to be the first study to compare survival curves for clinical recurrence, suicidal ideation, and suicide attempts with versus without long‐term lithium treatment of BD and MDD patients under the same conditions. These findings are particularly noteworthy given the typically high rates of recurrence and of suicidal behaviors in untreated or inadequately treated mood disorder patients [47]. The present findings accord with previous findings by Lepkifker and colleagues [48], who reported similar benefits of lithium treatment in both BD and MDD patients, with particularly robust effects in preventing episode recurrences in MDD.

The overall mean episode recurrence rate was 32.8% in the present sample without lithium treatment (Table 2). With BD, there was an expected 2.5‐times greater risk of new depression (70.0%) than of manic/mixed/hypomanic episodes (28.0%) [49]. Furthermore, the overall time in depressive phases of BD, and duration of depressive episodes are much greater than in mania or hypomania [47]. Moreover, reported recovery rates in BD also have been low, including syndromal recovery in only 48% of cases, symptomatic recovery in 26%, and functional recovery in 24% of patients during 12 months of treatment with a mood‐stabilizer [50]. Similarly, in MDD, recurrence rates can exceed 85% within a decade of an index episode, and approximately 50% have experienced relapse or recurrence within six months of remission when maintenance treatment was discontinued [46]. Also, the time to a new episode in MDD with antidepressant treatment continued was nearly 40 months versus only a year after treatment was discontinued [46]. The present findings indicate that lithium significantly improved all three of the outcomes considered (clinical recurrence, suicidal ideation and suicide attempt), including increased latency to their occurrence. They add to our previous finding that risk of psychiatric hospitalization in both BD and MDD patients was reduced with lithium in a similarly designed study [21].

The antisuicidal effects found in the present study are particularly important considering the high risk of suicidal behaviors with major affective disorders. Previous research found that up to 19% of BD patients died by suicide; lifetime prevalence of suicide attempts was 31% and one‐year prevalence was 8% [51, 52]. Cai et al. [53] reported that the prevalence of suicidal ideation in MDD patients for past month, past year, and past 2‐weeks were, respectively, 49.9%, 14.0% and 24.8%. In the current study, we found overall rates of 10.1% for suicidal ideation and 4.39% for suicide attempts during a year of lithium treatment. Among MDD patients when not given lithium, 64.8% had suicide ideation, compared to 13.6% when receiving lithium. Among BD patients when not receiving lithium, 36.3% experienced suicidal ideation compared to only 7.60% when given lithium. Among MDD patients not receiving lithium, 38.4% attempted suicide, compared to 6.40% with lithium treatment; among BD patients, suicide was attempted by 21.6% without lithium versus 2.90% with lithium (Table 3).

Previous investigations reported lifetime suicide attempt rates averaging 31.1% [27.9–34.3] (or 4.24%/year [3.78–4.70]) among BD patients [54]. A meta‐analysis of 22 studies involving 5647 patients found that suicide was 5.50‐fold (or 81.8%) less frequent during lithium treatment (0.159 vs. 0.875 deaths/100 patients/year), with a computed risk ratio in studies with rates off/on lithium of 8.85 ([4.12–19.1], *p* < 0.0001) [55]. Results of the present study confirm the efficacy of lithium on clinical recurrence and suicide risk in mood disorders. However, the generalizability of the present findings in other settings could be complicated by the unpredictable treatment adherence of patients and its relations to perceived benefits and adverse effects [56].

The present findings are consistent with previous research demonstrating lithium's superiority to placebo in reducing suicidal risk [20, 57]. In addition the present findings under clinically realistic conditions add support for the ability of lithium to reduce suicidal risks in both BD and MDD as well as to reduce risk of new episodes in both disorders (Tables 2 and 3) [58].

## 6 Limitations

A major limitation was the lack of “blinding” of participants or raters and reliance on clinical assessments rather than rating scales. The sample was relatively small, particularly for MDD patients, which may limit the statistical power of some comparisons. In addition, contributions of treatments provided clinically other than lithium were not ascertained. Finally, the reported outcomes may not generalize to other settings or clinical populations.

## 7 Conclusions

The present findings indicate that including lithium in the long‐term treatment of mood disorder patients was strongly associated with reduced risk of clinical recurrence, suicidal ideation and suicide attempts in patients diagnosed with either BD or MDD, as well as longer times to these outcomes. Notably, these effects associated with adding lithium treatment were similar or even greater with MDD than BD. The findings add support for the broad utility of lithium treatment in major affective disorders, including recent clinical guidelines recommending lithium as a primary treatment option for both BD and MDD patients at risk for suicide [12, 59]. Although further research is warranted to optimize treatment protocols, identify predictors of response, and elucidate specific mechanisms underlying lithium's antisuicidal properties, the present results underscore the continued clinical importance of lithium treatment for patients with major affective disorders.

## Author Contributions

**M.P.:** conceptualization; **M.P., I.B.:** methodology; **I.B., S.S., E.R. D.E.:** validation; **M.C., S.S.:** data analysis; **M.P., I.B., R.J.B.:** writing – original draft preparation; **M.P., I.B., D.L., R.J.B.:** writing, review and editing; **M.P.:** supervision and project administration.

## Disclosure

M. Pompili has received lecture and advisory board honoraria or has engaged in clinical trial activities with Allergan, Angelini Pharma, Boehringer‐Ingelheim, Fidia, Janssen, Lundbeck, Merck Sharp & Dohme, Otsuka, Pfizer, Recordati, Rovi, Teva, Newron, Neopharmed Gentili, Italfarmaco, and Viatris, and Viatris Corporations, all unrelated to the reported study. I. Berardelli has received consultant fees or lecture honoraria or participated in clinical trials with Angelini, Janssen, Lundbeck, and Rovi Corporations, all unrelated to the present study. Other authors have no affiliations or financial involvement with any commercial organization.

## Conflicts of Interest

The authors declare no conflicts of interest.

## Data Availability

The data that support the findings of this study are available on request from the corresponding author. The data are not publicly available due to privacy or ethical restrictions.

## References

[1] R. J. Baldessarini , G. L. Faedda , E. Offidani , et al., “Antidepressant‐Associated Mood‐Switching and Transition From Unipolar Major Depression to Bipolar Disorder: A Review,” Journal of Affective Disorders 148, no. 1 (2013): 129–135, 10.1016/j.jad.2012.10.033. 23219059

[2] M. Bauer and M. Gitlin , The Essential Guide to Lithium Treatment (Springer, 2016).

[3] G. S. Malhi , D. Gessler , and T. Outhred , “The Use of Lithium for the Treatment of Bipolar Disorder: Recommendations From Clinical Practice Guidelines,” Journal of Affective Disorders 217, no. 8 (2017): 266–280, 10.1016/j.jad.2017.03.052. 28437764

[4] J. Undurraga , K. Sim , L. Tondo , et al., “Lithium Treatment for Unipolar Major Depressive Disorder: Systematic Review,” Journal of Psychopharmacology 33, no. 2 (2019): 167–176, 10.1177/0269881118822161. 30698058

[5] F. Pelacchi , L. Dell'Osso , E. Bondi , et al., “Clinical Evaluation of Switching From Immediate‐Release to Prolonged‐Release Lithium in Bipolar Patients, Poorly Tolerant to Lithium Immediate‐Release Treatment: A Randomized Clinical Trial,” Brain and Behavior: A Cognitive Neuroscience Perspective 12, no. 3 (2022): e2485, 10.1002/brb3.2485. PMC8933786 35137572

[6] M. Pompili , C. Magistri , C. Mellini , G. Sarli , and R. J. Baldessarini , “Comparison of Immediate and Sustained Release Formulations of Lithium Salts,” International Review of Psychiatry 34, no. 7–8 (2022): 753–759, 10.1080/09540261.2022.2122706. 36705263

[7] E. Severus , M. J. Taylor , C. Sauer , et al., “Lithium for Prevention of Mood Episodes in Bipolar Disorders: Systematic Review and Meta‐Analysis,” International Journal of Bipolar Disorders 2, no. 12 (2014): 15–32, 10.1186/s40345-014-0015-8. 25530932 PMC4272359

[8] J. Tiihonen , A. Tanskanen , F. Hoti , et al., “Pharmacological Treatments and Risk of Readmission to Hospital for Unipolar Depression in Finland: A Nationwide Cohort Study,” Lancet Psychiatry 4, no. 7 (2017): 547–553, 10.1016/S2215-0366(17)30134-7. 28578901

[9] M. Carli , F. Weiss , G. Grenno , et al., “Pharmacological Strategies for Bipolar Disorders in Acute Phases and Chronic Management With a Special Focus on Lithium, Valproic Acid, and Atypical Antipsychotics,” Current Neuropharmacology 21, no. 4 (2023): 935–950, 10.2174/1570159X21666230224102318. 36825703 PMC10227916

[10] J. A. Fawcett , “Lithium Combinations in Acute and Maintenance Treatment of Unipolar and Bipolar Depression,” Journal of Clinical Psychiatry 64, no. Suppl 5 (2003): 32–37. 12720482. 12720482

[11] A. Fiorillo , G. Sampogna , U. Albert , et al., “Facts and Myths About the Use of Lithium for Bipolar Disorder in Routine Clinical Practice: An Expert Consensus Paper,” Annals of General Psychiatry 22, no. 1 (2023): 50–68, 10.1186/s12991-023-00481-y. 38057894 PMC10702081

[12] L. N. Yatham , S. H. Kennedy , S. V. Parikh , et al., “Canadian Network for Mood and Anxiety Treatments (CANMAT) and International Society for Bipolar Disorders (ISBD) 2018 Guidelines for the Management of Patients With Bipolar Disorder,” Bipolar Disorders 20, no. 2 (2018): 97–170, 10.1111/bdi.12609. 29536616 PMC5947163

[13] T. Miura , H. Noma , T. A. Furukawa , et al., “Comparative Efficacy and Tolerability of Pharmacological Treatments in the Maintenance Treatment of Bipolar Disorder: A Systematic Review and Network Meta‐Analysis,” Lancet Psychiatry 1, no. 5 (2014): 351–359, 10.1016/S2215-0366(14)70314-1. 26360999

[14] A. Cipriani , H. Pretty , K. Hawton , and J. R. Geddes , “Lithium in the Prevention of Suicidal Behavior and All‐Cause Mortality in Patients With Mood Disorders: Systematic Review of Randomized Trials,” American Journal of Psychiatry 162, no. 10 (2005): 1805–1819, 10.1176/appi.ajp.162.10.1805. 16199826

[15] B. Müller‐Oerlinghausen , B. Ahrens , and W. Felber , “Suicide‐Preventive and Mortality‐Reducing Effect of Lithium,” in Lithium in Neuropsychiatry, ed. M. Bauer , P. Grof , and B. Müller‐Oerlinghausen (CRC Press, 2013), 199–212.

[16] L. Tondo and R. J. Baldessarini , “Antisuicidal Effects in Mood Disorders: Are They Unique to Lithium?,” Pharmacopsychiatry 51, no. 5 (2018): 177–188, 10.1055/a-0596-7853. 29672801

[17] R. J. Baldessarini and L. Tondo , “Testing for Antisuicidal Effects of Lithium Treatment,” JAMA Psychiatry 79, no. 1 (2022): 9–10, 10.1001/jamapsychiatry.2021.2992. 34787652

[18] K. Dervic , L. Sher , H. C. Galfalvy , et al., “Antisuicidal Effect of Lithium in Bipolar Disorder: Is There an Age‐Specific Effect?,” Journal of Affective Disorders 341, no. 11 (2023): 8–11, 10.1016/j.jad.2023.08.107. 37619654

[19] A. Szmulewicz , A. Madenci , R. Ferguson , et al., “Estimating the Per‐Protocol Effect of Lithium on Suicidality in a Randomized Trial of Individuals With Depression or Bipolar Disorder,” Journal of Psychopharmacology 37, no. 6 (2023): 539–544, 10.1177/02698811231166460. 37039306 PMC13011873

[20] L. Tondo and R. J. Baldessarini , “Prevention of Suicidal Behavior With Lithium Treatment in Patients With Recurrent Mood Disorders,” International Journal of Bipolar Disorders 12, no. 1 (2024): 6–20, 10.1186/s40345-024-00326-x. 38460088 PMC10924823

[21] M. Pompili , I. Berardelli , S. Sarubbi , et al., “Lithium Treatment Versus Hospitalization in Bipolar Disorder and Major Depression Patients,” Journal of Affective Disorders 340, no. 11 (2023): 245–249, 10.1016/j.jad.2023.08.028. 37557990

[22] G. L. Faedda , L. Tondo , R. J. Baldessarini , T. Suppes , and M. Tohen , “Outcome After Rapid vs. Gradual Discontinuation of Lithium Treatment in Bipolar Disorders,” Archives of General Psychiatry 50, no. 6 (1993): 448–455, 10.1001/archpsyc.1993.01820180046005. 8498879

[23] G. M. Goodwin , “Recurrence of Mania After Lithium Withdrawal. Implications for the Use of Lithium in the Treatment of Bipolar Affective Disorder,” British Journal of Psychiatry 164, no. 2 (1994): 149–152, 10.1192/bjp.164.2.149. 8173817

[24] R. Kupka , E. Regeer , A. van Bergen , L. Tondo , and M. Bauer , “Lithium‐Discontinuation‐Induced Treatment Refractoriness Revisited,” International Journal of Bipolar Disorders 12, no. 1 (2024): 17–25, 10.1186/s40345-024-00339-6. 38750382 PMC11096143

[25] R. J. Baldessarini , L. Tondo , and J. Hennen , “Effects of Lithium Treatment and Its Discontinuation on Suicidal Behavior in Bipolar Manic‐Depressive Disorders,” Journal of Clinical Psychiatry 60, no. Suppl 2 (1999): 77–84. 10073392

[26] E. Kim , M. You , A. Pikalov , Q. Van‐Tran , and Y. Jing , “One‐Year Risk of Psychiatric Hospitalization and Associated Treatment Costs in Bipolar Disorder Treated With Atypical Antipsychotics: A Retrospective Claims Database Analysis,” BMC Psychiatry 11, no. 1 (2011): 6–15, 10.1186/1471-244X-11-6. 21214937 PMC3036592

[27] V. Caballer‐Tarazona , A. Zúñiga‐Lagares , and F. Reyes‐Santias , “Analysis of Hospital Costs by Morbidity Group for Patients With Severe Mental Illness,” Annals of Medicine 54, no. 1 (2022): 858–866, 10.1080/07853890.2022.2048884. 35318876 PMC8956305

[28] G. S. Malhi , E. Bell , T. Outhred , and M. Berk , “Lithium Therapy and Its Interactions,” Australian Prescriber 43, no. 3 (2020): 91–93, 10.18773/austprescr.2020.024. 32675910 PMC7358048

[29] G. Sesso , F. Bargnesi , F. Olzi , et al., “Efficacy and Safety of Lithium for Suicide and Suicide‐Related Behaviors in Youth: Review of the Literature,” Brain Sciences 14, no. 11 (2024): 1139–1156, 10.3390/brainsci14111139. 39595902 PMC11592384

[30] L. V. Kessing , “Why Is Lithium [Not] the Drug of Choice for Bipolar Disorder? Controversy Between Science and Clinical Practice,” International Journal of Bipolar Disorders 12, no. 1 (2024): 3–12, 10.1186/s40345-023-00322-7. 38228882 PMC10792154

[31] V. Singh , A. Kumar , and S. Gupta , “Mental Health Prevention and Promotion–a Narrative Review,” Frontiers in Psychiatry 13, no. 7 (2022): 898009, 10.3389/fpsyt.2022.898009. 35958637 PMC9360426

[32] Z. Nabi , J. Stansfeld , M. Plöderl , L. Wood , and J. Moncrieff , “Effects of Lithium on Suicide and Suicidal Behaviour: A Systematic Review and Meta‐Analysis of Randomised Trials,” Epidemiology and Psychiatric Sciences 31 (2022): e65–e76, 10.1017/S204579602200049X. 36111461 PMC9533115

[33] American Psychiatric Association (APA) , Diagnostic and Statistical Manual of Mental Disorders, 5th ed., text revision (DSM‐5‐TR); (American Psychiatric Publishing, 2022).

[34] K. Posner , M. A. Oquendo , M. Gould , B. Stanley , and M. Davies , “Columbia Classification Algorithm of Suicide Assessment (C‐CASA): Classification of Suicidal Events in the FDA'S Pediatric Suicidal Risk Analysis of Antidepressants,” American Journal of Psychiatry 164, no. 7 (2007): 1035–1043, 10.1176/ajp.2007.164.7.1035. 17606655 PMC3804920

[35] K. Posner , D. Brent , and C. Lucas , Columbia‐Suicide Severity Rating Scale (C‐SSRS) (Columbia University Medical Center, 2008).

[36] K. Posner , G. K. Brown , B. Stanley , et al., “Columbia‐Suicide Severity Rating Scale: Initial Validity and Internal Consistency Findings From Three Multisite Studies With Adolescents and Adults,” American Journal of Psychiatry 168, no. 12 (2011): 1266–1277, 10.1176/appi.ajp.2011.10111704. 22193671 PMC3893686

[37] M. M. Silverman , A. L. Berman , N. D. Sanddal , P. W. O'Carroll , and T. E. Joiner , “Rebuilding the Tower of Babel: A Revised Nomenclature for the Study of Suicide and Suicidal Behaviors. Part 1: Background, Rationale, and Methodology,” Suicide and Life‐threatening Behavior 37, no. 3 (2007): 248–263, 10.1521/suli.2007.37.3.248. 17579538

[38] M. M. Silverman , A. L. Berman , N. D. Sanddal , P. W. O'Carroll , and T. E. Joiner , “Rebuilding the Tower of Babel: A Revised Nomenclature for the Study of Suicide and Suicidal Behaviors. Part 2: Suicide‐Related Ideations, Communications, and Behaviors,” Suicide and Life‐threatening Behavior 37, no. 3 (2007): 264–277, 10.1521/suli.2007.37.3.264. 17579539

[39] K. Sim , W. K. Lau , J. Sim , M. Y. Sum , and R. J. Baldessarini , “Prevention of Recurrence in Adults With Major Depressive Disorder: Systematic Review and Meta‐Analyses of Controlled Trials,” International Journal of Neuropsychopharmacology 19, no. 2 (2015): pyv076, 10.1093/ijnp/pyv076. 26152228 PMC4772815

[40] K. N. Fountoulakis , “Refractoriness in Bipolar Disorder: Definitions and Evidence‐Based Treatment,” CNS Neuroscience & Therapeutics 18, no. 3 (2012): 227–237, 10.1111/j.1755-5949.2011.00259.x. 22070611 PMC6493614

[41] F. K. Goodwin and K. R. Jamison , Manic‐Depressive Illness: Bipolar Disorders and Recurrent Depression, 2nd ed. (Oxford University Press, 2007).

[42] C. H. Lin , Y. S. Chen , C. H. Lin , and K. S. Lin , “Factors Affecting Time to Rehospitalization for Patients With Major Depressive Disorder,” Psychiatry and Clinical Neurosciences 61, no. 3 (2007): 249–254, 10.1111/j.1440-1819.2007.01662.x. 17472592

[43] M. Tohen , E. Frank , C. L. Bowden , et al., “International Society for Bipolar Disorders (ISBD) Task Force Report on the Nomenclature of Course and Outcome in Bipolar Disorders,” Bipolar Disorders 11, no. 5 (2009): 453–473, 10.1111/j.1399-5618.2009.00726.x. 19624385

[44] F. Hardeveld , J. Spijker , R. De Graaf , W. A. Nolen , and A. T. Beekman , “Prevalence and Predictors of Recurrence of Major Depressive Disorder in the Adult Population,” Acta Psychiatrica Scandinavica 122, no. 3 (2010): 184–191, 10.1111/j.1600-0447.2009.01519.x. 20003092

[45] R. W. Licht , “Lithium: Still a Major Option in the Management of Bipolar Disorder,” CNS Neuroscience & Therapeutics 18, no. 3 (2012): 219–226, 10.1111/j.1755-5949.2011.00260.x. 22070642 PMC6493602

[46] R. J. Baldessarini , Chemotherapy in Psychiatry, 3rd ed. (Springer Press, 2013).

[47] R. J. Baldessarini , G. H. Vázquez , and L. Tondo , “Bipolar Depression: A Major Unsolved Challenge,” International Journal of Bipolar Disorders 8, no. 1 (2020): 1–14, 10.1186/s40345-019-0160-1. 31903509 PMC6943098

[48] E. Lepkifker , I. Iancu , N. Horesh , R. D. Strous , and M. Kotler , “Lithium Therapy for Unipolar and Bipolar Depression Among the Middle‐Aged and Older Adult Patient Subpopulation,” Depression and Anxiety 24, no. 8 (2007): 571–576, 10.1002/da.20273. 17133442

[49] R. H. Perlis , M. J. Ostacher , J. K. Patel , et al., “Predictors of Recurrence in Bipolar Disorder: Primary Outcomes From the Systematic Treatment Enhancement Program for Bipolar Disorder (STEP‐BD),” American Journal of Psychiatry 163, no. 2 (2006): 217–224, 10.1176/appi.ajp.163.2.217. 16449474

[50] A. C. Viguera , T. Whitfield , R. J. Baldessarini , et al., “Risk of Recurrence in Women With Bipolar Disorder During Pregnancy: Prospective Study of Mood Stabilizer Discontinuation,” American Journal of Psychiatry 164, no. 12 (2007): 1817–1824, 10.1176/appi.ajp.2007.06101639. 18056236

[51] M. Dong , S. B. Wang , Y. Li , et al., “Prevalence of Suicidal Behaviors in Patients With Major Depressive Disorder in China: Comprehensive Meta‐Analysis,” Journal of Affective Disorders 225, no. 1 (2018): 32–39, 10.1016/j.jad.2017.07.043. 28779680

[52] M. Dong , L. N. Zeng , L. Lu , et al., “Prevalence of Suicide Attempt in Individuals With Major Depressive Disorder: Meta‐Analysis of Observational Surveys,” Psychological Medicine 49, no. 10 (2019): 1691–1704, 10.1017/S0033291718002301. 30178722

[53] H. Cai , X. M. Xie , Q. Zhang , et al., “Prevalence of Suicidality in Major Depressive Disorder: Systematic Review and Meta‐Analysis of Comparative Studies,” Frontiers in Psychiatry 12, no. 9 (2021): 690130, 10.3389/fpsyt.2021.690130. 34603096 PMC8481605

[54] L. Tondo , M. Pompili , A. Forte , and R. J. Baldessarini , “Suicide Attempts in Bipolar Disorders: Comprehensive Review of 101 Reports,” Acta Psychiatrica Scandinavica 133, no. 3 (2016): 174–186, 10.1111/acps.12517. 26555604

[55] L. Tondo , J. Hennen , and R. J. Baldessarini , “Lower Suicide Risk With Long‐Term Lithium Treatment in Major Affective Illness: A Meta‐Analysis,” Acta Psychiatrica Scandinavica 104, no. 3 (2001): 163–172, 10.1034/j.1600-0447.2001.00464.x. 11531653

[56] M. Pompili , G. Serafini , A. Del Casale , et al., “Improving Adherence in Mood Disorders: The Struggle Against Relapse, Recurrence and Suicide Risk,” Expert Review of Neurotherapeutics 9, no. 7 (2009): 985–1004, 10.1586/ern.09.62. 19589049

[57] A. Cipriani , K. Hawton , S. Stockton , and J. R. Geddes , “Lithium in the Prevention of Suicide in Mood Disorders: Updated Systematic Review and Meta‐Analysis,” BMJ (Clinical research ed.) 346, no. 6 (2013): f3646, 10.1136/bmj.f3646. 23814104

[58] S. K. Sarai , H. M. Mekala , and S. Lippmann , “Lithium Suicide Prevention: Brief Review and Reminder,” Innovations in Clinical Neuroscience 15, no. 11–12 (2018): 30–32. 30834169. PMC6380616 30834169

[59] N. Verdolini , D. Hidalgo‐Mazzei , L. Del Matto , et al., “Long‐Term Treatment of Bipolar Disorder Type I: A Systematic and Critical Review of Clinical Guidelines With Derived Practice Algorithms,” Bipolar Disorders 23, no. 4 (2021): 324–340, 10.1111/bdi.13040. 33354842

